# Discordancy Partitioning for Validating Potentially Inconsistent Pharmacogenomic Studies

**DOI:** 10.1038/s41598-017-15590-4

**Published:** 2017-11-09

**Authors:** J. Sunil Rao, Hongmei Liu

**Affiliations:** 1 Division of Biostatistics, Department of Public Health Sciences, University of Miami, Florida, USA; 2 Sylvester Comprehensive Cancer Center, University of Miami, Florida, USA

## Abstract

The Genomics of Drug Sensitivity in Cancer (GDSC) and Cancer Cell Line Encyclopedia (CCLE) are two major studies that can be used to mine for therapeutic biomarkers for cancers of a large variety. Model validation using the two datasets however has proved challenging. Both predictions and signatures do not consistently validate well for models built on one dataset and tested on the other. While the genomic profiling seems consistent, the drug response data is not. Some efforts at harmonizing experimental designs has helped but not entirely removed model validation difficulties. In this paper, we present a partitioning strategy based on a data sharing concept which directly acknowledges a potential lack of concordance between datasets and in doing so, also allows for extraction of reproducible novel gene-drug interaction signatures as well as accurate test set predictions. We demonstrate these properties in a re-analysis of the GDSC and CCLE datasets.

## Introduction

It’s now widely believed that cancers are far more heterogeneous than once thought - that in fact, they represent a myriad of different diseases with varying biological determinants rather than a single entity whose effective treatment will rely on some over-arching theoretical construct of our understanding of the disease. To this end, high throughput pharmacogenomic screening of small molecules and other compounds has the potential to implicate new drug leads (or drug combinations) that can be used for more personalized treatments.

A typical pharmacogenomic workflow involves characterizing interesting compounds for dose-response effects on cancer cell lines, and then doing functional genomic characterization in additional screens. Of interest is the elucidation of therapeutic biomarkers whose patterns might be predictive of a compound’s activity against a particular cancer cell line.

Given the complexity of such assays, the necessity of proper validation is paramount. Given a fresh set of data generated from the same workflow, one should be able to demonstrate both accurate predictions of drug activity as well as reproducibility of therapeutic genomic signatures.

## The GDSC and CCLE datasets

The opportunity to study validation of models arose with the generation of two major pharmacogenomic datasets - the Genome Drug Sensitivity in Cancer (GDSC) project^1,2^ and the Cancer Cell Line Encyclopedia (CCLE) project^3^. Both represent large-scale studies in which experimental and approved drug compounds were screened against panels of molecularly characterized cancer cell lines.

A number of recent studies looked at the 15 drugs and 706 cell lines which were in common between the two studies^4–6^. One study acted as a training dataset and the other the test dataset. Focus was put initially on examining concordance of drug response profiles and genomic profiles across datasets, then later moving to reproducibility of detected drug-gene interactions. Some concerning results were published^5^ that reported poor reproducibility of drug response profiles but high concordance of genomic profiles. A more stringent analysis was then done^4^ which used more biologically grounded analytical considerations yielding improved consistency of pharmacological data but still only reaching 67% of evaluable compounds. Given that the ultimate goal of such studies is to detect novel drug-gene interactions, successful validation also implies that training data models of drug-gene interactions should yield low test set prediction errors of drug response *and* reproducibility of therapeutic genomic signatures on the test dataset. *It should be noted however*, *that in general developing accurate signatures* (*those with true drug*-*gene interactions*) *does not always imply low prediction errors*. Overfit models are also known to predict well (and these might also yield reproducible signatures) - however the test set error differences between overfit and accurate more sparse models is usually not large^7^.

Some additional studies have been carried out by adding a third new drug screening dataset to the mix to see if improved concordance could be achieved^8–10^. In all cases, the GDSC-CCLE discordancies could not be completely resolved. For instance, a third independent dataset called the Genentech Cell Line Screening Initiative (gSCI)^10^ was generated. Improved agreement appears to be present between gSCI and CCLE, and concordance with GDSC was weaker. However, careful reading of this paper shows that there are still issues with the improved agreement that was found. The proportion of shared genomic features found is low, association values are not compared (meaning the actual magnitudes and signs of the effects are not displayed), and the elastic net model they used was only fit once and all non-zero estimates were regarded as candidate biomarkers. Typically these models need proper setting of tuning parameters which are found by methods like cross-validation. Cross-validation in itself can be unstable and thus it’s more robust to repeat the process many times and look at a more rigorous definition of a candidate biomarker in terms of repeated detection over many iterations of cross-validation. In another study, a third independent dataset from the Institute for Molecular Medicine Finland (FIMM)^9^ was generated which shared a much more similar experimental protocol to CCLE than GDSC did. This dataset had a significantly higher level of consistency with CCLE than with GDSC although correlations across cell lines were markedly lower than between cell lines. Note that only 26 cell lines were shared while CCLE and GDSC share 268 cell lines, so even though the conclusions reached by the authors about the need to carefully standardize experimental protocols may be correct, a selection bias cannot be ruled out. Still the improved results does not completely resolve the discordancy issue. Recently, even a detailed re-analyses of an updated (and larger version) of the GDSC and CCLE data using many additional suggestions from the research community did not result in significantly improved validation findings^11^. The same trends of low concordance of drug profiles, high concordance of genomic profiles and poor detection of novel drug-gene interactions remain - and these patterns are not constant across drugs. So while new datasets and improved harmonization of protocols is certainly important, because validation is a multi-faceted concept, discordancy issues of one type or another may still remain - although our hope is over time, these will continue to be mitigated.

In this paper we take a more agnostic view: we assume that discordancies *may* exist for a given drug, and we build this assumption into our modeling framework. This is followed up with using shrinkage estimation of model parameters which can *zero out* discordancies *if in fact they are small*. That way, we can handle both scenarios - drugs that are concordant and those that might not be (whatever the underlying reasons). Our approach also can achieve both low test set prediction errors and detect reproducible gene-drug interactions - many of which we found to be novel. We develop a **discordancy partitioning** approach using the data sharing strategy^12,13^ across datasets which can easily be extended to more than two datasets.

## Methods

**Figure 1:**
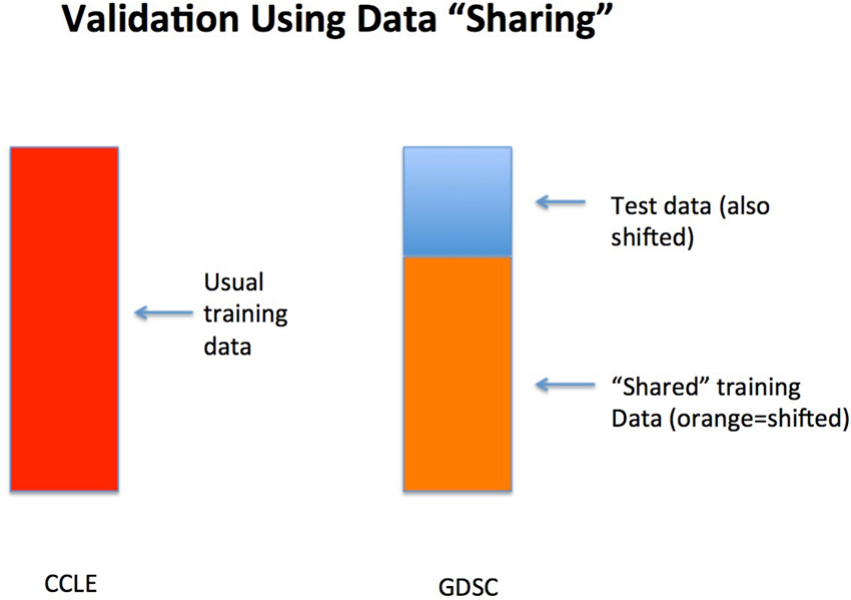
Schematic of data sharing for the combined GDSC-CCLE analysis.

Figure 1 presents a schematic of our analysis strategy. We use all of the CCLE dataset and a *portion* of the GDSC dataset as training. The remainder of the GDSC dataset will be withheld from modeling and used purely for evaluating test set prediction errors. In order to have proper representation of all tissue types, we ensure that the portion of GDSC used for modeling contains cell lines sampled at random from all tissue types.

The goal of the analysis is to identify therapeutic genomic signatures for each drug (and potentially further by cancer type). The response variable *y* comes from the drug response assay and has typically been taken to be the *log*(*IC*50) value or the drug-specific area under the curve (AUC) value from the dose-response curve. The predictors **x**, come from the molecular assays and include, mutation status of known cancer genes, gene expression profiles, copy number variations and cancer tissue types. We consider the following two model formulations:

Formulation 1:

1 $$\begin{array}{l}{y}_{i}={{\boldsymbol{\beta }}}^{T}{{\bf{x}}}_{i}+{\varepsilon }_{i},i\in G\\ {y}_{i}={({\boldsymbol{\beta }}+{\boldsymbol{\delta }})}^{T}{{\bf{x}}}_{i}+{\varepsilon }_{i},i\in C,\end{array}$$

where ∈ *G* or ∈ *C* most generally represent two independent datasets, but here can represent cell line membership in GDSC or CCLE datasets respectively. The model dimensions are ${y}_{i}\in {\mathbb{R}}$, ${{\bf{x}}}_{i}\in {{\mathbb{R}}}^{p}$, ${\boldsymbol{\beta }}\in {{\mathbb{R}}}^{p}$, ${\boldsymbol{\delta }}\in {{\mathbb{R}}}^{p}$. The errors ${\varepsilon }_{i}\in {\mathbb{R}}$ are identically and independent distributed with zero mean and finite variance. Model (1) can be generalized to more than two datasets and can also allow for multiple drug responses where ${{\bf{y}}}_{i}\in {{\mathbb{R}}}^{q}$. We will report on a new model fitting strategy for the multivariate model elsewhere.

Model (1) formally partitions the common genomic effects across datasets (***β***) from the potential dataset discordancies (***δ***). A version of this was called data shared lasso^12^ which used lasso estimation^14^ to induce sparsity in ***β*** and ***δ***. An earlier version of this approach traces to the data enriched linear regression^13^. We use a different constraint on the model parameters by using a generalized version of the elastic net^15^. Specifically, we add an additional *L*_2_ constraint on the model parameters which allows finding sparse signal with potential grouped effects which represent correlated genomic features. The following penalized optimization function is used,

2 $$\begin{array}{rcl}(\hat{{\boldsymbol{\beta }}},\hat{{\boldsymbol{\delta }}}) & = & {\rm{\arg }}\,{\rm{\min }}\,\frac{1}{2}\,\sum _{i\in G}\,{({y}_{i}-{{\bf{x}}}_{i}^{T}{\boldsymbol{\beta }})}^{2}+\sum _{i\in C}\,{({y}_{i}-{{\bf{x}}}_{i}^{T}({\boldsymbol{\beta }}+{\boldsymbol{\delta }}))}^{2}\\  &  & +{\lambda }_{1}\,(\sum _{j=1}^{p}\,{\omega }_{j}|{\beta }_{j}|+r\,\sum _{j=1}^{p}\,{\psi }_{j}|{\delta }_{j}|)+{\lambda }_{2}\,({\Vert {\boldsymbol{\beta }}\Vert }_{2}^{2}+r{\Vert {\boldsymbol{\delta }}\Vert }_{2}^{2}),\end{array}$$

where *λ*_1_, *λ*_2_ > 0 are tuning parameters. The elastic net estimators were suggested to be used to construct the weights, where ${\omega }_{j}={|{\hat{\beta }}_{j}^{enet}|}^{-1}$ and ${\psi }_{j}={|{\hat{\delta }}_{j}^{enet}|}^{-1}$, because they are $\sqrt{n}$-consistent estimators and can guarantee variable selection consistency in (2), i.e. the true covariates in the model can be identified^16^. We let $r=1/\sqrt{2}$ as recommended^12^. Ten-fold cross validation is used to optimize *λ*_1_ and *λ*_2_ from a pre-specified set of values.

To avoid a lack of a potential invariance due to the choice of the reference group in (1), an alternate formulation of the model can be made as represented in Formulation 2:

Formulation 2:

3 $$\begin{array}{l}{y}_{i}={({\boldsymbol{\beta }}+{{\boldsymbol{\delta }}}_{G})}^{T}{{\bf{x}}}_{i}+{\varepsilon }_{i},i\in G\\ {y}_{i}={({\boldsymbol{\beta }}+{{\boldsymbol{\delta }}}_{C})}^{T}{{\bf{x}}}_{i}+{\varepsilon }_{i},i\in C,\end{array}$$

where ${{\boldsymbol{\delta }}}_{G}\in {{\mathbb{R}}}^{p}$ are discrepancy parameters of G, ${{\boldsymbol{\delta }}}_{C}\in {{\mathbb{R}}}^{p}$ are discrepancy parameters of C. Here ***β*** represents a baseline effect that is shared across datasets. Formulations 1 and 2 are two different ways to model discrepancies between datasets. However, the two models are potentially interchangeable. More specifically, denote as ***β***_1_ the ***β*** in Formulation 1 and ***β***_2_ the ***β*** in Formulation 2. Then one can show that ***β***_1_ = ***β***_2_ + ***δ***_*G*_ and ***δ*** = ***δ***_*C*_ − ***δ***_*G*_. We will restrict most of our attention to Formulation 1 since fewer parameters need to be estimated and fitted models are generally more stable as a result especially when sample size is small.

### Simulation Design

In order to study performance of our proposed approach, we first conduct a simulation study where we know what the true level of dataset discordancy is and compare it against alternative strategies. Specifically, we consider the following simulation design.

Training data were generated from model (1) where **x**_*i*_ were i.i.d. from *N*_*p*_(**0**, **Σ**) with *p* = 1000 and Σ(*i*, *j*) = 0.75^|*i* − *j*|^, and *ε*_*i*_ was i.i.d. from *N*(0, 1). The autoregressive covariance structure is to mimic high correlations between neighboring genes (i.e. this could be thought of as genes on the same biological pathway for instance). Also since not all 1000 features are likely to be correlated with the response variable, we assumed both ***β*** and ***δ*** were sparse. To mimic grouped effects in genomic data, non-zero elements in ***β*** were distributed in 16 blocks with block size 3, and the non-zero values were randomly drawn from *N*(1.5, 1). Moreover, we assumed that there are discordances in both non-zero effect genes and zero-effect genes. Twenty indices of non-zero entries and 10 indices of zero entries of ***β*** were randomly selected which constituted the indices of non-zero elements of ***δ***, and the non-zero values were randomly drawn from *N*(0, 0.5).

In the training dataset, we let the sample size of dataset G increase from 100 to 400 and the sample size of dataset C be fixed at 300. We also drew a test dataset with sample size 100 from the upper equation of model (1), i.e. from the same distribution as G dataset. We applied model (1) to training datasets G and C, and calculated the mean prediction error on test data using the shared models. The prediction performances were compared to other candidate models: individual model built on dataset G, individual model built on dataset C and individual model built on merged datasets of G and C. The simulation was repeated 200 times.

Note that the same input training datasets G and C can also be fitted by Formulation 2 and test set mean prediction error can be calculated. Moreover, as mentioned above, model parameters from Formulation 2 can be transformed into parameters in Formulation 1. We will not present the results here.

### Analysis of GDSC and CCLE Datasets

#### Data Preprocessing

We used the drug measure AUC as a response variable. There are 15 drugs in common between the GDSC and CCLE datasets. While 706 cell lines are also shared between the two datasets and have associated genomic feature data, only a subset of these cell lines have AUC values in both datasets. By further removing cell lines with different SNP identity^11^ and cell lines of which the number of missing values in feature data is greater than 10000, we get a range of 77–274 cell lines per drug (median = 88; mean = 162 cell lines).

The shared genomic feature data includes mutation status of 63 cancer genes, copy number variations of 24960 genes, 16150 gene expression profiles and 23 tissue types. Genomic features whose missing rate was larger than 0.3 were filtered. The remaining feature data had less than <0.1% missing values which were imputed by feature means. We standardized each covariate vector and drug response vector to have mean 0 and variance 1.

#### Data Analysis Strategy

In analysis of prediction accuracy, we applied model (1) to the shared portions of GDSC and CCLE datasets. Because the shared subset data only have a limited number of cell lines and many cell lines miss the copy number variation data, we excluded copy number variations from the feature set of the model. The data also went though a quality control by removing a few number of outliers.

A random sample of 20% GDSC dataset were withheld as test dataset. Similar to the design in simulation study, we let sample size of the GDSC training dataset vary to see effects of increased sharing. Thus in the first scenario, the CCLE dataset and a random sample of 70% of the 80% GDSC dataset were used as training data. In the second scenario, the CCLE dataset and the 80% GDSC dataset were used as training data. In each scenario, we used sure independence screening (SIS)^17^ to reduce the number of covariates to a moderate size, where 2000 features most correlated with the response variable were selected respectively in *each* training CCLE and training GDSC dataset. A union of the two sets of selected features were used as input variables. Then model (1) was fitted to the screened training dataset. Mean prediction error for the 20% GDSC test dataset using either the shared GDSC model or shared CCLE model was calculated. This process was repeated 200 times to account for the variability of random sampling. We compared the mean prediction errors from the shared models against following methods: i) using the training GDSC dataset alone (this represents a gold standard subject to sample size limitations when discordancies exist between the datasets), ii) using the training CCLE dataset alone, iii) merging the training GDSC and training CCLE datasets as one common training dataset.

Then we switched the roles of GDSC and CCLE datasets and conducted a similar prediction accuracy analysis. More specifically, a random sample of 20% CCLE dataset were withheld as test dataset. We let sample size of the CCLE training dataset vary. In the first scenario, a random sample of 70% of the 80% CCLE dataset and the GDSC dataset were used as training data. In the second scenario, the 80% CCLE dataset and the GDSC dataset were used as training data. The rest are similar to previous prediction analysis where 20% GDSC dataset were withheld as test dataset.

For signature validation analysis, we ran a shared analysis using both datasets in full for the 15 drugs in common. All cell lines in GDSC and CCLE were used, and copy number variations were included into the feature set of the model because of the increased sample size available for analysis. Sure independence screening was used to reduce the number of covariates to a moderate size in a same way as in previous prediction accuracy analysis. Then model (1) was fitted to the screened GDSC and CCLE datasets. We examined the estimated ***β*** and ***δ*** values in order to determine how many genomic markers reliably reproduced their effects across datasets, and how many effects were *washed away* because of dataset discordancies.

Covariates with non-zero estimates were determined to be predictors associated with the drug response variable. This procedure was repeated 200 times for each drug to assess the stability of covariates when applying ten-fold cross validation for tuning parameters selection in equation (2). A variable list was built for each drug. It consists of all covariates that appeared in any of the 200 runs along with a frequency that a feature appears and an average coefficient given to that feature over the runs it appears. The average coefficient was used to assess effect size of a feature to the drug response variable. Here, the most significant predictors associated with the drug response variable are defined as those with an effect size ±2 s.d. from the mean and a frequency rate ≥80%. We also fit Formulation 2 to the same screened GDSC and CCLE datasets. Estimates of ***β*** + ***δ***_*G*_ and ***β*** + ***δ***_*C*_ were examined.

## Results

### Simulation

**Figure 2:**
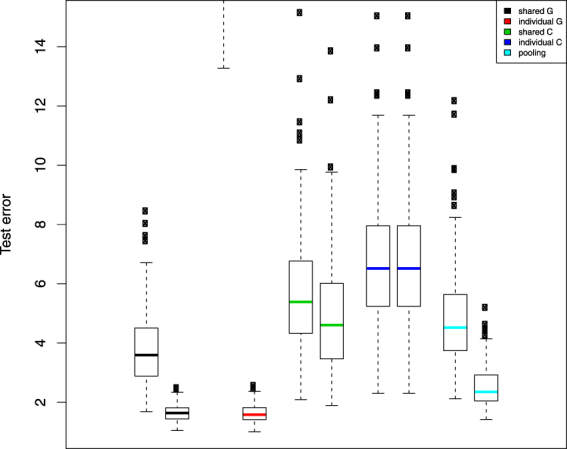
Simulation test set error boxplot pairs by analysis method. Left hand member of the pair represents a training set sample size of 100 and the right hand member, a training set sample size of 400. Test set sample sizes are always set a 300. Black represents predictions generated by using $\hat{{\boldsymbol{\beta }}}$ only estimated from the data sharing model across G and C; red represents predictions generated from a model using only dataset G; green represents predictions using $\hat{{\boldsymbol{\beta }}}+\hat{{\boldsymbol{\delta }}}$ only from the data sharing model across G and C; blue represents predictions generated from a model using only dataset C; and finally light blue, predictions from a model pooling datasets G and C.

Prediction results in the simulation study are summarized in Fig. 2. When sample sizes are small, the shared G model outperformed all other methods and individual G model has much larger test errors than other methods do (which exceeds the range of the plot). Note that non-overlapping interquartile ranges of boxplots signify clearly statistically significant differences. Also, interquartile ranges that may overlap but do not overlap with another boxplot’s median value also indicate statistically significant differences in prediction error performance. As sample size increases, the individual G model serves as a gold standard (as the test data were from this model), and more importantly the shared G model performs clearly as well as this gold standard. Since there are non-negligible disparities between data G and C due to the ***δ*** in our simulations, the shared/individual C model and individual aggregated model never beat the shared G model in all cases.

**Figure 3:**
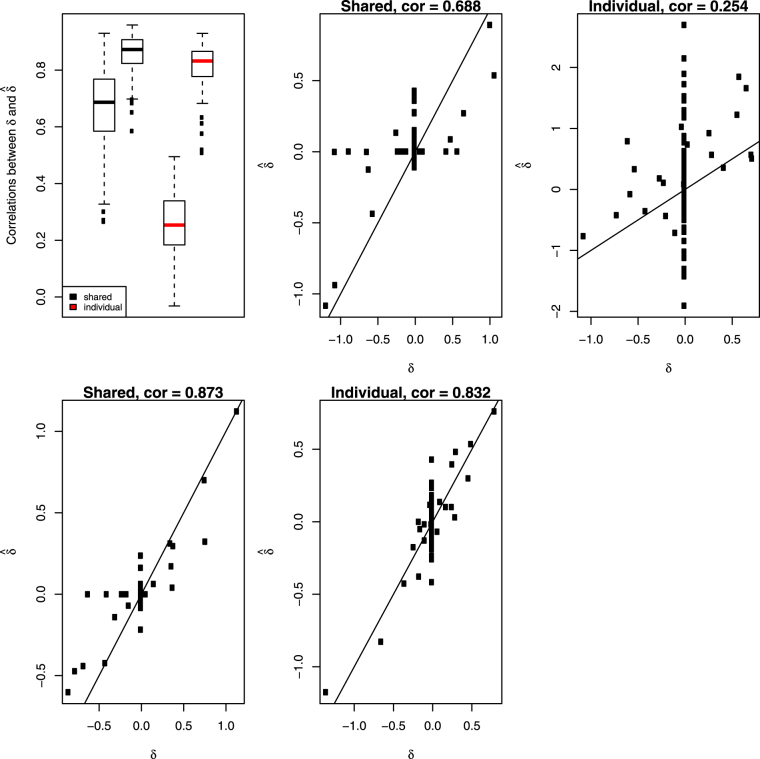
Top left panel: Simulation correlations between estimated ***δ*** and the true *δ* across the 200 runs of the simulation for the shared model (black) and extracted from fitted individual models (red). The side by side boxplots represents the two different sample sizes in the simulation. All other panels show the scatterplots of estimated values of ***δ*** versus true values at the median correlations from the boxplots in the top left panel.

**Figure 4:**
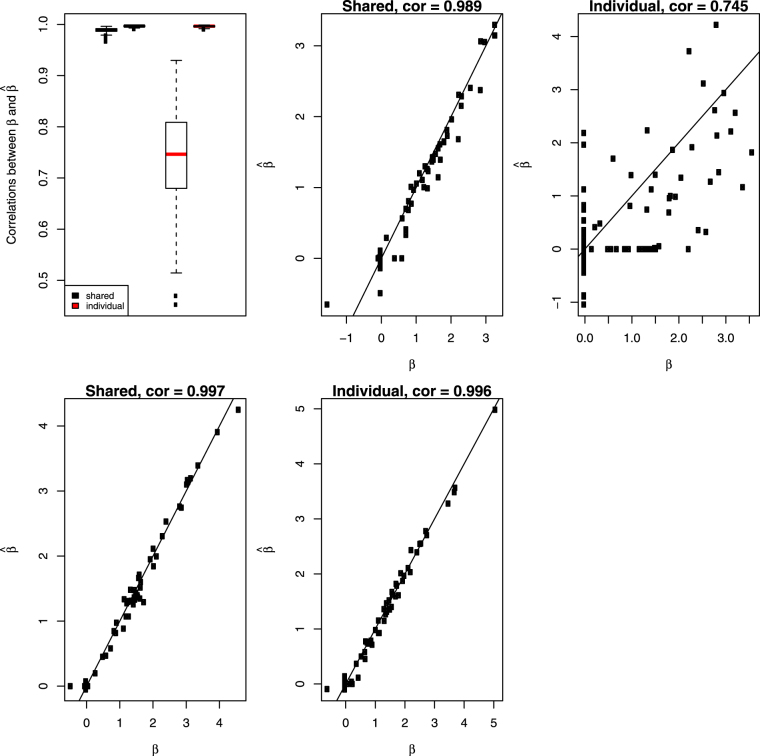
Top left panel: Simulation correlations between estimated ***β*** and the true *β* across the 200 runs of the simulation for the shared model (black) and extracted from fitted individual models (red). The side by side boxplots represents the two different sample sizes in the simulation. All other panels show the scatterplots of estimated values of ***β*** versus true values at the median correlations from the boxplots in the top left panel.

Correlations of $\hat{{\boldsymbol{\delta }}}$ from model (1) with the true ***δ*** in 200 simulations were calculated and present in boxplot of Fig. 3. For comparison, we subtracted ${\hat{{\boldsymbol{\beta }}}}_{G}$ (estimate of ***β*** from individual G model) from ${\hat{{\boldsymbol{\beta }}}}_{C}$ (estimate of ***β*** from individual C model), then calculated its correlation with the true ***δ***. Not surprisingly, the shared method designed for estimating dataset discrepancies produced much higher correlations than alternative method did, see Fig. 3. We also calculated correlations of $\hat{{\boldsymbol{\beta }}}$ from model (1) with the true ***β*** in 200 simulations and compared them to the correlations between ${\hat{{\boldsymbol{\beta }}}}_{G}$ and the true ***β***. Results were summarized in Fig. 4. Again, model (1) produced much higher correlations than individual G model did when sample size is small. As sample size largely increases, individual G model becomes the gold standard and model (1) is as good as this gold standard.

### GDSC and CCLE Datasets

**Figure 5:**
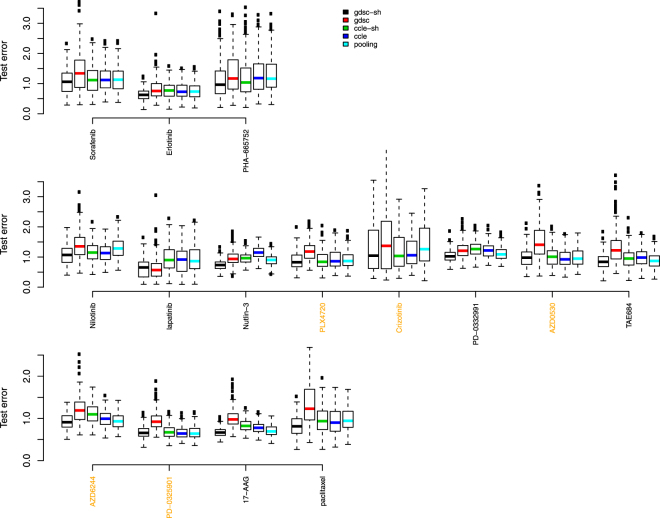
Test error rate boxplots by drug when 70% of 80% GDSC dataset were used in training data and 20% GDSC dataset were reserved as test data. Black represents predictions based on data sharing estimates for ***β*** alone; red for predictions from a model using GDSC alone; green for predictions from a data sharing model using estimates of ***β*** + ***δ***; blue for predictions from a model using CCLE alone, and light blue, predictions from a pooled GDSC + CCLE model.

**Figure 6:**
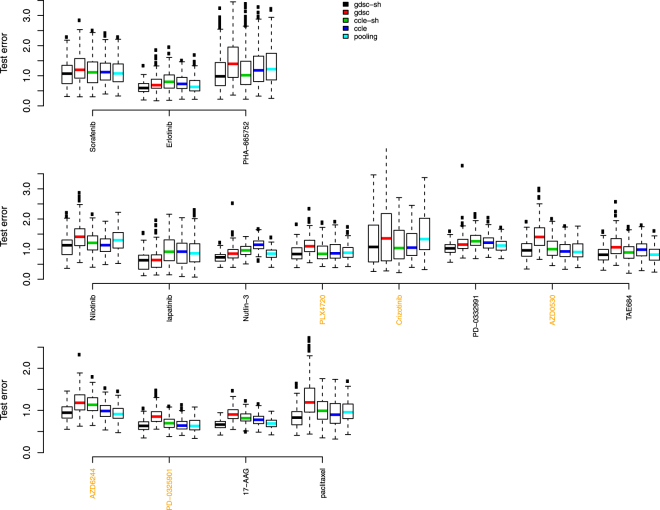
Test error rates by drug when 80% GDSC were used in training data and 20% GDSC dataset were reserved as test data. Black represents predictions based on data sharing estimates for ***β*** alone; red for predictions from a model using GDSC alone; green for predictions from a data sharing model using estimates of ***β*** + ***δ***; blue for predictions from a model using CCLE alone, and light blue, predictions from a pooled GDSC + CCLE model.

Figure 5 shows side-by-side boxplots (over the 200 random splits of the GDSC dataset) of test set error rates for the common 15 drugs when 70% of 80% GDSC dataset (scenario 1) was used in training data. Figure 6 show results when all 80% GDSC dataset (scenario 2) was used in training data. The changes, however, are negligible between the two scenarios. This is reasonable given the limited change in sample size. More specifically, given a median sample size of 88, the sample size was only increased by 21 in scenario 2. Highlighted in yellow, are the names of drugs where other groups had found reasonable concordance between the two drug response datasets^4^ (Pearson correlation > 0.45).

We used following criteria to compare the side-by-side boxplots. If interquartile ranges of two boxplots do not overlap, there is a statistically meaningful difference in measures of central location between distributions. When two interquartile ranges overlap, we calculate the distance between the medians (DBM) as a percentage of overall visible spread (OVS), i.e. BDM/OVS, where OVS is the distance between the lower quarter of one box and the higher quarter of another box. For a sample size of 100, if this percentage is over 20%, there is likely a statistically meaningful difference between the two boxplots. We opt for this conservative approach rather than reporting multiple p-values and then having to correct for multiple testing because it’s a more intuitive way to relate inference to the boxplots themselves.

Some striking conclusions are evident. Among the compared methods, it is easy to see that the reduction in sample size by using the GDSC training data alone (the red boxplots) has a clear deleterious effect. Typical modeling would use the CCLE dataset alone (the dark blue boxplots). Table 1 shows the BDM/OVS values for boxplots with respect to the shared GDSC model and CCLE alone model for scenario 2. For drugs with reasonable concordance, the shared method does not provide much in way of test set error reductions. This is exactly what should happen. However, for 7 out of the other 10 drugs, the shared method clearly produces the most accurate models in terms of test set error rates. The 7 drugs are Erlotinib, PHA-665752, lapatinib, Nutlin-3, PD-0332991, TAE684 and 17-AAG. For the rest 3 drugs, the shared method is as good as or slightly better than the CCLE alone method.

**Table 1:** Statistical metrics to compare test error rates from shared GDSC model (numerator) and CCLE alone model (denominator) in scenario 2 when portion of GDSC dataset was withheld as test data.

Drug	min	lower quarter	median	upper quarter	max	BDM/OVS
Sorafenib	$\tfrac{0.307}{0.394}$	$\tfrac{0.741}{0.857}$	$\tfrac{1.07}{1.12}$	$\tfrac{1.347}{1.419}$	$\tfrac{2.18}{2.2}$	0.075
Erlotinib	$\tfrac{0.196}{0.221}$	$\tfrac{0.477}{0.578}$	$\tfrac{0.593}{0.729}$	$\tfrac{0.742}{0.945}$	$\tfrac{1.042}{1.467}$	0.292
PHA-665752	$\tfrac{0.221}{0.324}$	$\tfrac{0.672}{0.81}$	$\tfrac{0.982}{1.182}$	$\tfrac{1.438}{1.652}$	$\tfrac{2.546}{2.881}$	0.204
Nilotinib	$\tfrac{0.362}{0.488}$	$\tfrac{0.819}{0.92}$	$\tfrac{1.129}{1.131}$	$\tfrac{1.302}{1.34}$	$\tfrac{2.027}{1.928}$	0.004
lapatinib	$\tfrac{0.117}{0.092}$	$\tfrac{0.332}{0.532}$	$\tfrac{0.632}{0.915}$	$\tfrac{0.799}{1.196}$	$\tfrac{1.298}{2.041}$	0.328
Nutlin-3	$\tfrac{0.392}{0.747}$	$\tfrac{0.602}{1.016}$	$\tfrac{0.726}{1.144}$	$\tfrac{0.82}{1.271}$	$\tfrac{1.12}{1.644}$	0.624
PLX4720	$\tfrac{0.384}{0.386}$	$\tfrac{0.676}{0.699}$	$\tfrac{0.836}{0.86}$	$\tfrac{1.045}{1.155}$	$\tfrac{1.488}{1.805}$	0.049
Crizotinib	$\tfrac{0.256}{0.392}$	$\tfrac{0.576}{0.785}$	$\tfrac{1.071}{1.05}$	$\tfrac{1.798}{1.516}$	$\tfrac{3.464}{2.453}$	−0.023
PD-0332991	$\tfrac{0.563}{0.722}$	$\tfrac{0.894}{1.036}$	$\tfrac{1.025}{1.214}$	$\tfrac{1.151}{1.372}$	$\tfrac{1.486}{1.815}$	0.395
AZD0530	$\tfrac{0.339}{0.33}$	$\tfrac{0.764}{0.755}$	$\tfrac{0.959}{0.922}$	$\tfrac{1.182}{1.155}$	$\tfrac{1.727}{1.734}$	−0.093
TAE684	$\tfrac{0.3}{0.285}$	$\tfrac{0.641}{0.778}$	$\tfrac{0.815}{0.981}$	$\tfrac{0.987}{1.174}$	$\tfrac{1.453}{1.744}$	0.312
AZD6244	$\tfrac{0.554}{0.536}$	$\tfrac{0.819}{0.857}$	$\tfrac{0.946}{0.987}$	$\tfrac{1.085}{1.119}$	$\tfrac{1.46}{1.436}$	0.138
PD-0325901	$\tfrac{0.347}{0.408}$	$\tfrac{0.549}{0.56}$	$\tfrac{0.634}{0.641}$	$\tfrac{0.73}{0.74}$	$\tfrac{0.981}{0.996}$	0.037
17-AAG	$\tfrac{0.417}{0.485}$	$\tfrac{0.59}{0.689}$	$\tfrac{0.666}{0.777}$	$\tfrac{0.741}{0.861}$	$\tfrac{0.947}{1.115}$	0.411
paclitaxel	$\tfrac{0.409}{0.321}$	$\tfrac{0.661}{0.696}$	$\tfrac{0.83}{0.9}$	$\tfrac{0.972}{1.165}$	$\tfrac{1.407}{1.736}$	0.14

**Figure 7:**
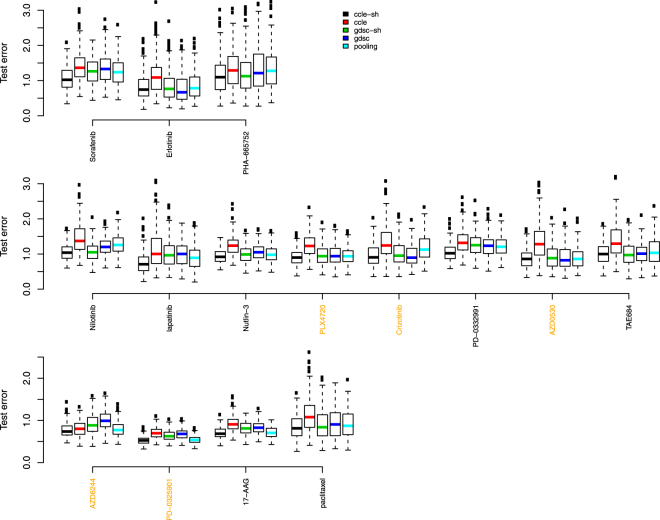
Test error rate boxplots by drug when 70% of 80% CCLE dataset were used in training data and 20% CCLE dataset were reserved as test data. Black represents predictions based on data sharing estimates for ***β*** alone; red for predictions from a model using GDSC alone; green for predictions from a data sharing model using estimates of ***β*** + ***δ***; blue for predictions from a model using CCLE alone, and light blue, predictions from a pooled GDSC + CCLE model.

**Figure 8:**
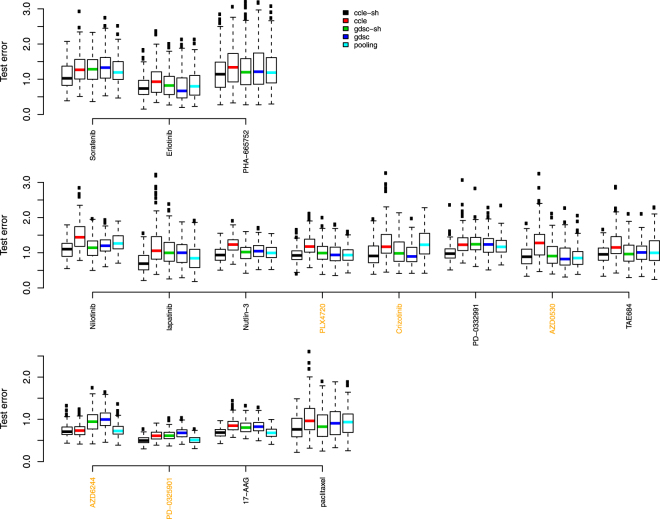
Test error rates by drug when 80% CCLE dataset were used in training data and 20% CCLE dataset were reserved as test data. Black represents predictions based on data sharing estimates for ***β*** alone; red for predictions from a model using GDSC alone; green for predictions from a data sharing model using estimates of ***β*** + ***δ***; blue for predictions from a model using CCLE alone, and light blue, predictions from a pooled GDSC + CCLE model.

Figures 7 and 8 show results where 20% CCLE dataset were reserved as test data, and Table 2 shows the BDM/OVS values for boxplots with respect to the shared CCLE model and GDSC alone model for scenario 2. Conclusions are similar to the analysis where 20% GDSC dataset were reserved as test data. Now, the shared method clearly produces the most accurate models in terms of test set error rates for 2 out of the 5 drugs with reasonable concordance and for 7 out of the other 10 discordant drugs. They include Sorafenib, Nilotinib, lapatinib, Nutlin-3, PD-0332991, AZD6244, PD-0325901, 17-AAG and paclitaxel.

**Table 2:** Statistical metrics to compare test error rates from shared CCLE model (numerator) and GDSC alone model (denominator) in scenario 2 when portion of CCLE dataset was withheld as test data.

Drug	min	lower quarter	median	upper quarter	max	BDM/OVS
Sorafenib	$\tfrac{0.387}{0.528}$	$\tfrac{0.8234}{1.032}$	$\tfrac{1.025}{1.329}$	$\tfrac{1.372}{1.615}$	$\tfrac{2.074}{2.449}$	0.384
Erlotinib	$\tfrac{0.150}{0.196}$	$\tfrac{0.569}{0.468}$	$\tfrac{0.736}{0.668}$	$\tfrac{0.970}{1.037}$	$\tfrac{1.493}{1.882}$	−0.145
PHA-665752	$\tfrac{0.273}{0.273}$	$\tfrac{0.770}{0.857}$	$\tfrac{1.141}{1.214}$	$\tfrac{1.486}{1.745}$	$\tfrac{2.498}{2.967}$	0.075
Nilotinib	$\tfrac{0.548}{0.603}$	$\tfrac{0.899}{1.048}$	$\tfrac{1.104}{1.200}$	$\tfrac{1.267}{1.367}$	$\tfrac{1.794}{1.761}$	0.205
lapatinib	$\tfrac{0.211}{0.290}$	$\tfrac{0.514}{0.726}$	$\tfrac{0.690}{1.001}$	$\tfrac{0.923}{1.230}$	$\tfrac{1.457}{1.878}$	0.435
Nutlin-3	$\tfrac{0.508}{0.520}$	$\tfrac{0.786}{0.892}$	$\tfrac{0.936}{1.047}$	$\tfrac{1.097}{1.204}$	$\tfrac{1.554}{1.582}$	0.266
PLX4720	$\tfrac{0.444}{0.352}$	$\tfrac{0.807}{0.772}$	$\tfrac{0.925}{0.938}$	$\tfrac{1.055}{1.159}$	$\tfrac{1.418}{1.691}$	0.038
Crizotinib	$\tfrac{0.389}{0.415}$	$\tfrac{0.716}{0.749}$	$\tfrac{0.910}{0.897}$	$\tfrac{1.194}{1.154}$	$\tfrac{1.907}{1.726}$	−0.029
PD-0332991	$\tfrac{0.511}{0.512}$	$\tfrac{0.849}{1.016}$	$\tfrac{0.974}{1.235}$	$\tfrac{1.112}{1.405}$	$\tfrac{1.448}{1.960}$	0.47
AZD0530	$\tfrac{0.332}{0.311}$	$\tfrac{0.687}{0.653}$	$\tfrac{0.887}{0.821}$	$\tfrac{1.102}{1.135}$	$\tfrac{1.689}{1.795}$	−0.146
TAE684	$\tfrac{0.333}{0.326}$	$\tfrac{0.788}{0.812}$	$\tfrac{0.955}{1.01}$	$\tfrac{1.135}{1.192}$	$\tfrac{1.569}{1.719}$	0.136
AZD6244	$\tfrac{0.435}{0.457}$	$\tfrac{0.638}{0.853}$	$\tfrac{0.706}{0.997}$	$\tfrac{0.818}{1.149}$	$\tfrac{1.062}{1.587}$	0.568
PD-0325901	$\tfrac{0.3}{0.408}$	$\tfrac{0.446}{0.587}$	$\tfrac{0.496}{0.68}$	$\tfrac{0.566}{0.752}$	$\tfrac{0.702}{0.984}$	0.601
17-AAG	$\tfrac{0.425}{0.492}$	$\tfrac{0.604}{0.728}$	$\tfrac{0.686}{0.826}$	$\tfrac{0.76}{0.922}$	$\tfrac{0.967}{1.209}$	0.44
paclitaxel	$\tfrac{0.217}{0.329}$	$\tfrac{0.586}{0.636}$	$\tfrac{0.761}{0.906}$	$\tfrac{1.025}{1.182}$	$\tfrac{1.487}{1.889}$	0.242

**Figure 9:**
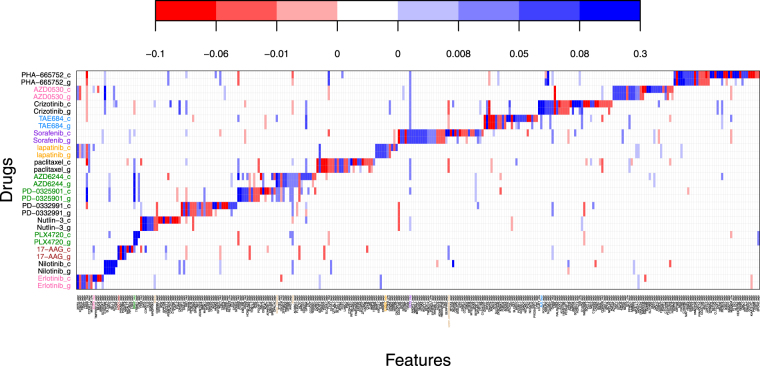
Signature validation plot based on GDSC (g) and CCLE (c) datasets using Formulation 1. The y-axis shows the different drugs under study. The x-axis shows the markers which had significant effects. Blue coloring indicates a positive estimated effect; red a negative estimated effect. White indicates an estimated null effect.

Figure 9 shows a very interesting plot of signature validation for the 15 drugs in common. The y-axis shows results from the fit of model (1) on the GDSC data (lowercase g) and the CCLE data (lowercase c). The x-axis depicts a subset of genomic effects with at least one highly significant non-zero effect across the 15 drugs. The body of the plot shows the estimated biomarker effect sizes. For g-drugs, the $\hat{{\boldsymbol{\beta }}}$ values are plotted. For c-drugs, the $(\hat{{\boldsymbol{\beta }}}+\hat{{\boldsymbol{\delta }}})$ values are plotted. Blue colors indicate markers which predict drug sensitivity, red the opposite - the darker the color, the more intense the effect. Other genomic predictors not plotted showed no highly significant non-zero estimated effects for any of the 15 drugs. Shown in color are the known drug-marker associations^6^. Clearly we do an excellent job of recovering those. We find all of the ones previously found except for the Nutlin-3-MDM2 expression interaction. The association pattern of Crizotinib-HGF expression interaction was recovered. It however was not identified as highly significant based on previously described inclusion rule and thus is not shown in Fig. 9. What is more interesting is how many new reproducible markers the data sharing strategy finds. For each drug, new bands of markers are discovered. What’s also noticeable is that the bands do not entirely overlap between g and c. These areas without overlap are markers where the dataset discordancies were large enough to wash away true effects $(|{\delta }_{j}|\gg |{\beta }_{j}|)$ such that they were not detected as reproducible. Closer examination of the particular drugs reveals that the washing out effect is happening in those drugs where discordancies were previously established and much less so where concordance was found. *This is exactly what the theory would have predicted*.

One can dig further into the signature validation plot. For instance, population stratifications can be made to better identify only those patients who may provide a higher likelihood of a favorable drug responses. Take nilotnib as an example. Here, having increased gene expression in C15orf26, SLC4A1, MPO, IGLL1, and APOL4 (i.e. the reproducible markers shaded dark blue) would be predictive of a favorable drug response. In addition, this was accentuated for thyroid cell lines as compared to the other cancer cell lines. On the other hand, for PLX4720, having the BRAF mutation and TRIM51 expression present, would predict a favorable response *as long as no NRAS mutation was present*. Other interesting patterns like this can be gleaned for each drug.

The horizontal banding is clearly what is most visible initially. However, one can also look vertically at specific genomic markers and find interesting information. For example, with the KRAS mutation, the only drug which shows strong reproducible predicted sensitivity to its presence is PD-0325901 - a MEK inhibitor. Many other drugs are predicted to encounter a resistance effect as indicated by the red c-g pairs along the KRAS column. In fact, recent evidence suggests that PD-0325901 used in conjunction with dacomitinib may be of use when KRAS mutations are present^18^.

**Figure 10:**
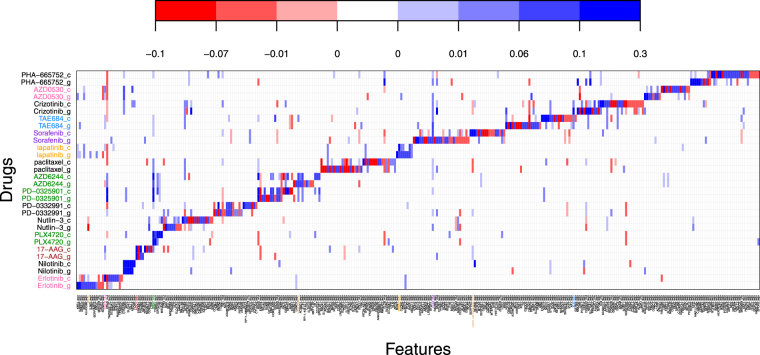
Signature validation plot based on GDSC (g) and CCLE (c) datasets using Formulation 2. The y-axis shows the different drugs under study. The x-axis shows the markers which had significant effects. Blue coloring indicates a positive estimated effect; red a negative estimated effect. White indicates an estimated null effect.

Figure 10 shows a signature validation plot based on model Formulation 2. Focusing on a specific discoveries illuminates how to reconcile Figs 9 and 10. Take the TP53-mut biomarker and the drug Nutlin-3 for instance. Both Formulation 1 and Formulation 2 fits (Figs 9 and 10 respectively), show bright red colorations for both datasets CCLE and GDSC. This would indicate a gene-drug interaction that validated with both model Formulations. Take the neighboring biomarker BAX-expr for the same drug: in Formulation 1, the interaction appears solid blue for both datasets. For Formulation 2, it’s slightly darker blue for GDSC than CCLE. This simply implies that by Formulation 2, the fitted effects across the two datasets are very similar with GDSC having a slightly stronger difference from baseline than CCLE. Translating back to Formulation 1, this means that the concordancy effect estimate dominates the discordancy estimate which is exactly what we see. Other similar inferences can be made looking at different gene-drug interactions between the two Formulation plots. However, contrast this to the BRCA2 mutation. It does not show up at all on Fig. 9. Thus one may conclude that no detectable signal was associated with this marker for any drug. This can be confirmed by examining Fig. 10 which shows the signature validation plot using Formulation 2. Here BRCA2 does show and does indicate a smaller reproducible effect for Nutlin-3. However the coloring is identical indicating that the estimated values of ***δ***_*C*_ and ***δ***_*g*_ are either estimated very similarly in magnitude (or both estimated near zero). The difference between the two model Formulations is attributable to differences in model tuning and to the stringency of the inclusion rules that are used.

## Discussion

We have demonstrated that reproducible signal can in fact be partitioned from signal due to dataset discordancy using data sharing strategies. The most exciting thing may in fact be that we found new reproducible biomarkers for *every* drug in the analysis, whereas others have only been able to do so for about half of the drugs^11^. Furthermore, patient populations can be partitioned based on their reproducible biomarker profiles to more precisely predict a favorable response to a drug. Additionally, we have shown that test set prediction error rates are markedly lower when using discordancy partitioning models and very much follow established theory. Thus while experimental challenges may still exist in order to better standardize protocols, it’s likely datasets will never be completely concordant. Discordancy partitioning approaches like what we have presented can adapt easily to varying degrees of discordancy to produce more accurate assessments of validation.

As for the methodology itself, a few more remarks are in order. With regards to estimation, it should be noted that we are not saying that the combined elastic net/lasso estimation technique is the only one that could be used here. In fact we developed our own new modeling strategy based on a generalized finite mixture of regressions model which can be used to find interesting new patterns of therapeutic biomarkers^19^. However, even this new modeling strategy can be embedded within a data sharing strategy for validation purposes. The reason we chose to illustrate results with the elastic net/lasso approach is because it’s a more widely known approach for estimating sparse genomic models.
